# What you see is not always what you get—MRI-based ganglionic eminence volumetry challenges subjective assessment in CNS anomalies

**DOI:** 10.1007/s00330-026-12636-y

**Published:** 2026-05-21

**Authors:** Marlene Stuempflen, Patric Kienast, Victor U. Schmidbauer, Michael Weber, Athena Taymourtash, Johannes Tischer, Tim Dorittke, Julia Binder, Daniela Prayer, Gregor Kasprian

**Affiliations:** 1 Department of Biomedical Imaging and Image-guided Therapy, Medical University of Vienna, Vienna, Austria; 2 Computational Imaging Research Lab, Department of Biomedical Imaging and Image-Guided Therapy, Medical University of Vienna, Vienna, Austria; 3 Department of Obstetrics and Feto-Maternal Medicine, Medical University of Vienna, Vienna, Austria; 4 Comprehensive Center for Clinical Neuroscience and Mental Health, Medical University of Vienna, Vienna, Austria

**Keywords:** CNS, Fetal MRI, Fetal neurodevelopment, Ganglionic eminence, Magnetic resonance imaging

## Abstract

**Objectives:**

To compare prenatal imaging-based subjective assessment of the ganglionic eminence (GE) on fetal MRI with three-dimensional volumetric analysis in patients with structural central nervous system anomalies.

**Materials and methods:**

This retrospective study investigated 17 fetuses (undergoing 20 fetal MRIs, mean gestational age 26.3 weeks, SD 3.3, range 21.7–33.4 weeks) with enlarged GE based on subjective assessment of fetal neuroimaging experts and concurrent structural brain anomalies. Three-dimensional volumetry of super-resolution MRI was performed and compared to age-matched neurotypical controls (94 fetuses, 100 MRIs, mean age 27.2 weeks, SD 3.6, range 21.7–34.0 weeks).

**Results:**

Among 20 MR examinations, in only 5 cases (25%) GE hyperplasia was confirmed, while 12 (60%) were found to show normal GE volumes. In 3 (15%), GE volumes were smaller compared to controls. Most patients (80%) were found to have increased total brain volume, while only 30% had an increased intracranial volume—ventriculomegaly (75%) seemingly being the most common underlying cause. Brain parenchyma volume was enlarged in only 20%. No correlation was found between GE volumes and volumes of 10 other substructures of the fetal head in pathological cases.

**Conclusion:**

The study suggests the unreliability of expert visual assessment of GE size, despite excellent examination conditions, emphasizing the need for three-dimensional volumetric GE measurements. Specifically in patients with structural brain anomalies, quantitative fetal neuroimaging serves as an emerging tool to identify abnormalities in GE size and complement current diagnostic techniques by objectifying subjective impressions.

**Key Points:**

***Question*** *To evaluate the reliability of expert visual assessment of ganglionic eminence size in fetal MRI compared to super-resolution brain volumetry.*

***Findings*** *Expert visual assessment and 2D fetal brain biometry were insufficient to provide correct volumetric quantification of the ganglionic eminence in the investigated patient collective.*

***Clinical Relevance*** *This study highlights the value of volumetric quantification of the ganglionic eminence in fetal MRI to provide an objective, reproducible and valid quantitative tool for assessment of the fetal brain, specifically in the presence of CNS anomalies.*

**Graphical Abstract:**

**Figure:**
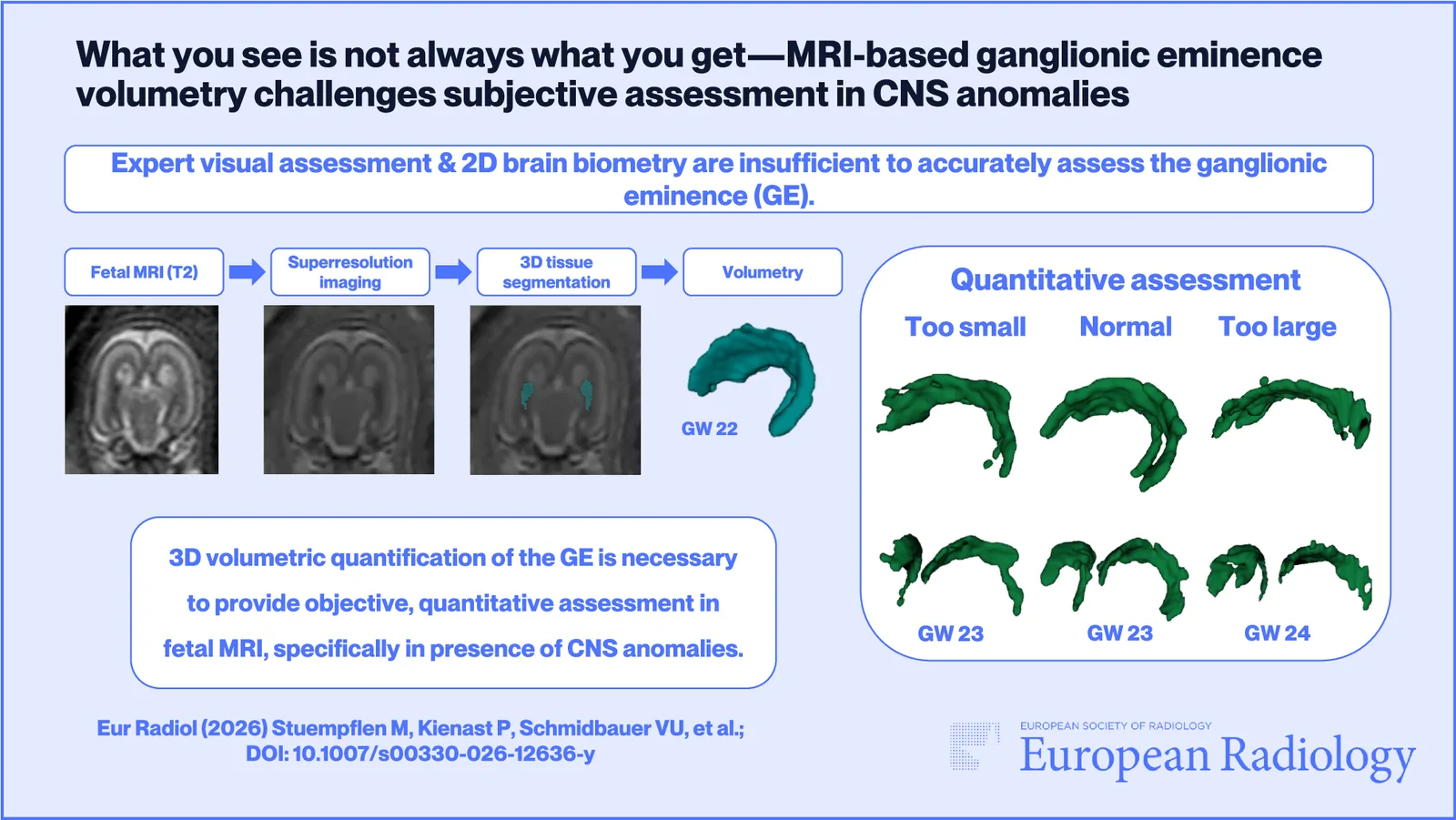

## Introduction

The ganglionic eminence (GE) is a transient fetal brain structure arising in the ventral portion of the telencephalon adjacent to the lateral ventricles [1]. Progenitor cells derived from the GE differentiate into inhibitory interneurons and projection neurons migrating to various substructures of the fetal brain, including the cortex [2–5].

Structural anomalies within the GE may lead to abnormal migration of progenitor cells harboring potentially severe consequences, including severe developmental delay or therapy-resistant epilepsy [6]. The structural appearance and etiology of these anomalies may vary and can be categorized into groups: While microstructural anomalies are commonly associated with epilepsy [6], GE cavitations commonly result in reduced opercularization, reduced gyration or even lissencephaly [7, 8]. Disorders of cellular energy generation and preservation, including mitochondriopathies such as pyruvate dehydrogenase deficiency, may also result in structural GE anomalies [9]. Various genetic disorders may result in structural anomalies of the GE, including disorders of neonatal proliferation such as hemimegalencephaly, tuberous sclerosis and other mTORopathies [10–12]. Volumetric changes of the GE, specifically GE enlargement, were found to be associated with various other structural anomalies, including macrocephaly, abnormal transient brain layering, and unilateral polymicrogyria [8, 12].

Lastly, due to its strong vascularization, the GE is the most common site of neonatal hemorrhage in preterm infants and intraventricular hemorrhage in fetuses [13, 14].

The GE can be visualized in ultrasound from the late first trimester onwards, presenting with median echogenicity and is usually assessed with two-dimensional measurements [15]. During the second and third trimesters, neuroimaging experts can visualize the GE in 90% of cases [16]. In fetal MRI, the GE is often assessed subjectively based on its appearance in T2-weighted images [17]. However, due to drastic changes in shape and size of the GE, subjective assessment poses the risk of missing subtle anomalies and requires profound experience in fetal neuroimaging. A recent study analyzed the GE based on three-dimensional segmentation and volumetric measurements, highlighting the importance of quantification of this fetal brain structure [18].

This quantitative fetal neuroimaging study aimed to assess the GE in patients with structural CNS anomalies: Applying super-resolution fetal MRI and three-dimensional segmentation of the GE, patients with subjectively enlarged GE were assessed and compared to controls—firstly, to assess whether subjective assessment in patients with structural CNS anomalies is feasible and secondly, to identify potential structural anomalies that complicate subjective assessment of the GE.

## Materials and methods

### Participants

In this retrospective study, we examined women with singleton pregnancies who were seen at a tertiary referral center from January 2010 to July 2023. Informed consent was obtained, and this study was conducted in accordance with the Helsinki Declaration of 2013 and with approval of the ethics committee (EK 1585/2021). Institutional patient records were screened for women undergoing fetal MRI, with reports including the diagnosis of an enlarged GE based on subjective assessment by two experienced fetal neuroimaging experts in consensus, and corresponding information regarding maternal medical history. Following initial inclusion of all identified patients and generation of super-resolution reconstruction of fetal MRI, the inclusion and exclusion criteria were applied: inclusion for further analysis required the availability of detailed second trimester ultrasound report including gestational age (as determined by ultrasound and given in gestational weeks (GWs) and days post menstruation), high-quality super-resolution reconstruction of the fetal head, and imaging data of the whole body of the fetus to assess confounding comorbidities as well as adherence to the standard requirements with the current International Society of Ultrasound in Obstetrics & Gynecology Practice Guidelines for fetal MRI despite the retrospective nature of this study [19]. Patients with cystic or hemorrhagic alterations in the GE were excluded from this study.

The selected patients with subjectively enlarged GE were allocated to the “pathological” group (regardless of additional concurrent structural anomalies) and compared to a control group of patients without structural CNS anomalies or other confounding comorbidities affecting fetal neurodevelopment (e.g., cardiac defects, placental anomalies, fetal growth restriction), which was part of a study collective previously published [18]. In the control group, patients with minor structural anomalies were included in this study due to our institutional review board's ethical considerations, which prohibited the inclusion of an entirely normal reference cohort. To our knowledge, none of the structural anomalies observed in the control group have been previously shown to be associated with abnormal GE development. An overview of all observed structural anomalies in both investigated groups is provided in Supplementary Table 1.

### Fetal MRI

All MRI scans were clinically indicated and referred by a prenatal ultrasound expert or fetal medicine specialist. Informed consent was obtained prior to the MRI examination. Fetal MRI scans were performed on 1.5-T (Philips Ingenia/Intera) and 3-T (Philips Achieva) scanners. Women were imaged in a supine or left recumbent position without the application of contrast agent or sedation. Imaging of the fetal head included T2-weighted sequences (slice thickness 2.0–4 mm, echo time = 100– 140 ms, field of view = 200–230 mm, in-plane resolution 0.62/0.62–1.17/1.17 mm) in three orthogonal planes. The fetal head and body were investigated, but the quantitative analysis of this collective focused on the T2-weighted images of the fetal head, while imaging of the fetal body and maternal structures was used to rule out patients with confounding comorbidities. Additionally, two-dimensional measurements were performed, which included fronto-occipital and biparietal diameters of the fetal brain and skull as well as head circumference calculated by one rater (M.S.) using the calculator tool provided by Kyriakopoulou et al [20] and the resulting percentiles were extracted.

### Postprocessing

Super-resolution imaging postprocessing was based on the fetal brain atlas provided by Gholipour et al [21] and conducted as previously published in another study [18]: Following acquisition of T2-weighted sequences of the fetal head in three orthogonal planes without fetal movement artifacts, imaging data were denoised, in-plane super-resolution was generated, and automatic brain masking was conducted. This yielded a single 0.5 mm isotropic volume using combined slice-wise motion correction and a volumetric super-resolution algorithm [18, 22]. The resulting super-resolution data were subjected to a quality assessment by two raters (M.S.—5+ years of experience in fetal imaging, G.K.—15+ years of experience in fetal imaging) using a 5-point image quality scale. Cases that did not meet high-quality standards (score > 2) were excluded from the analysis. Volumetric assessment of the fetal brain was performed using super-resolution reconstruction of available fetal MRI imaging data rather than the raw fetal MR imaging data, as previous studies have demonstrated that super-resolution imaging provides higher spatial resolution, as well as increased three-dimensional volume accuracy, geometric reliability, and improved biometry [23, 24]. Semi-automated atlas-based segmentation of the super-resolution reconstructions and manual correction were performed for cases with sufficient image quality. A total of eleven fetal brain structures were assessed: cortex, subcortical parenchyma (including the subcortical layers between the cortical plate and subventricular zone), deep gray nuclei and thalamus, periventricular zone (including subventricular and ventricular zones), corpus callosum, hippocampus, GE, cerebellum, brainstem, inner, and outer cerebrospinal fluid (CSF) spaces. Values were calculated for total brain volume (TBV, including inner CSF spaces), brain parenchyma volume (BPV, excluding inner CSF spaces), white matter volume (WMV), and intracranial volume (ICV). Manual correction was performed by two raters blinded for gestational age (M.S., P.K.—5+ years of experience in fetal imaging) using the open-source application ITK-SNAP [25].

### Rater-based assessment, segmentation, and evaluation

Subjective assessment of enlarged GE volume was based on consensus between two raters (D.P.—20+ years of experience in fetal imaging; G.K.—15+ years of experience in fetal imaging). Quality control of imaging data was performed by two raters in consensus (M.S.—5+ years of experience in fetal imaging, G.K.—15+ years of experience in fetal imaging). Super-resolution reconstruction of fetal MRI data and semi-automated tissue segmentation of selected cases were performed by a computational scientist (J.T.). Standardized two-dimensional measurements of the fetal brain and skull were performed by one rater (M.S.—5+ years of experience in fetal imaging). Manual correction of tissue segmentations was performed by two raters blinded for gestational age (M.S. and P.K.—both 5+ years of experience in fetal imaging). Statistical analysis was performed by a postdoctoral biostatistician (M.W.—25+ years of experience in biostatistics).

### Statistical analysis

Volumetric analysis of fetal brain structures was based on gestational age. As 7 patients were examined twice at different gestational ages, a random effect for patient identity was added. Imaging data generated on 1.5- and 3-T scanners were analyzed indifferently, as has been previously shown to be feasible. *p*-values used for significance were defined as *p* < 0.05.

For a subset of 14 patients (one for each included GW), the GE was segmented by two raters (M.S., P.K.) independently, and compartment-based mean Hausdorff distance and Sørensen-Dice coefficient were computed using in-house Python scripts to assess reproducibility of the segmentations. Volumetric measurements were extracted and visualized by generating scatterplots for each analyzed substructure and specific combinations of substructures (e.g., TBV).

Nonlinear regression was used to model the effect of gestational age on the analyzed brain substructures, using only controls. Based on these regressions, 95% confidence intervals for individual measures were calculated. Firstly, each case was classified as too small (if its measure was below the lower limit of the 95% CI), normal (if its measure was between the 95% CI), or too high (if its measure was above the upper limit of the 95% CI). Secondly, each measure was transformed into a *z*-score. These *z*–scores were correlated between different volumes of all assessed brain structures. Furthermore, percentiles of two-dimensional fetal brain measurements, including fronto-occipital and biparietal diameters of the fetal brain and skull, were correlated to three-dimensional volumetric measurements of TBV and ICV using a Spearman-Rho correlation.

All calculations were performed using IBM SPSS Statistics for Windows, Version 28.

## Results

### Patients and demographics

**Fig. 1:**
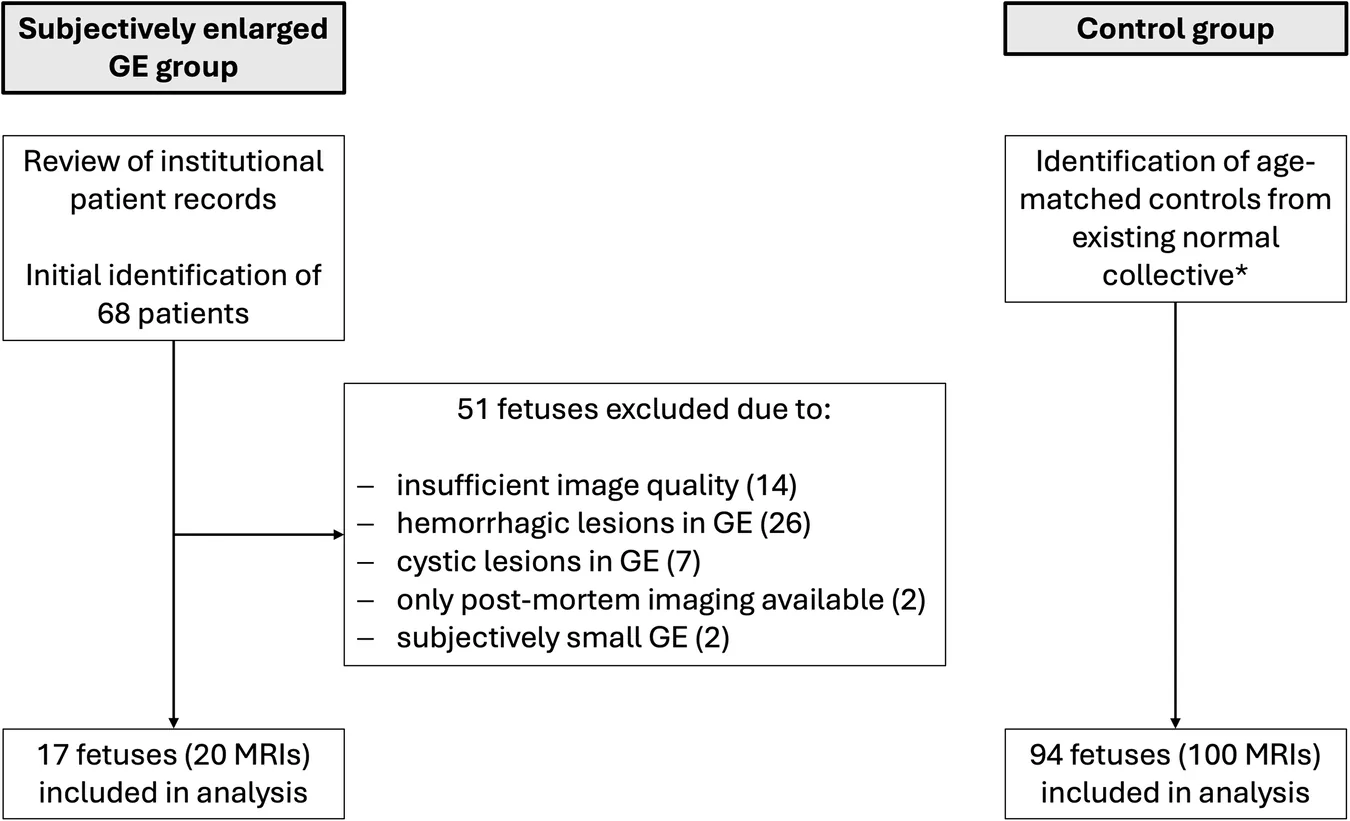
Flowchart depicting the patient selection process. GE , ganglionic eminence. *as previously published by Stuempflen M et al, UOG, 2023, https://doi.org/10.1002/uog.26232

An initial search of institutional patients records yielded 68 patients (86 MRI scans) with structurally abnormal GE (Fig. 1). During patient assessment, 51 women undergoing 66 MRI scans had to be excluded for the following reasons: insufficient super-resolution image quality—despite good source data quality (14 patients, 21 MRIs), hemorrhagic lesions in GE (26 patients, 28 MRIs), cystic lesions in GE (7 patients, 9 MRIs), only postmortem MRI available (2 patients, 6 MRIs), subjectively small GE (2 patients, 2 MRIs). Thus, a total of 17 patients (20 MRIs, mean gestational age 26.3 GWs (GW), SD + /−3.3, range 21.7-33.4 GW) with GE—rated as “enlarged” by two independent expert raters—were analyzed in this study.

Additionally, a total of 94 controls (100 MRIs) were selected from a pre-existing collective [18] of cases without structural CNS anomalies or confounding comorbidities (e.g., cardiac defects, fetal growth restriction) based on gestational age (mean gestational age 27.2 GW, SD+/− 3.6, range 21.7–34.0 weeks). The demographic data of both groups is summarized in Table 1.

**Table 1:** Demographics

	Subjectively enlarged GE group	Control group
Sample size	17 (20)	94 (100)
Gestational age (GW)	26.3 (3.3)	27.2 (3.6)
Maternal age (years)	31.4 (6.1)	31.6 (5.2)
Fetal sex (female)	47.1%	33.0%

Sample size given in number of fetuses (number of scans), ages given in mean (standard deviation). *GW* gestational weeks

A table highlighting the final diagnoses of both groups is provided as Supplementary Table 1.

### Volumetric results

Assessment of intra- and interrater correlation of segmentations was based on evaluation of Hausdorff distance and Sørensen-Dice-coefficients calculated for a subset of 14 patients (one for each investigated GW) and analyzed segmentations of the GE performed by two raters, independently (M.S. vs. M.S. and M.S. vs. P.K.). The results are summarized in Table 2. The high mean Sørensen-Dice-coefficients for intra- and interrater measurements indicate a very high level of correlation among the analyzed segmentations.

**Table 2:** Assessment of intra- and interrater-correlation: Hausdorff distance and Sørensen-Dice-coefficient values for a subset of 14 patients (one for each investigated gestational week) based on ganglionic eminence segmentations, segmented by two raters, independently

	Intrarater correlation	Interrater correlation
Hausdorff distance	2.515 (1–10.989)	2.88 (0.866–6.021)
Sørensen-Dice-coefficient	0.919 (0.812–0.964)	0.893 (0.708–0.995)

Values given as mean (range)

**Fig. 2:**
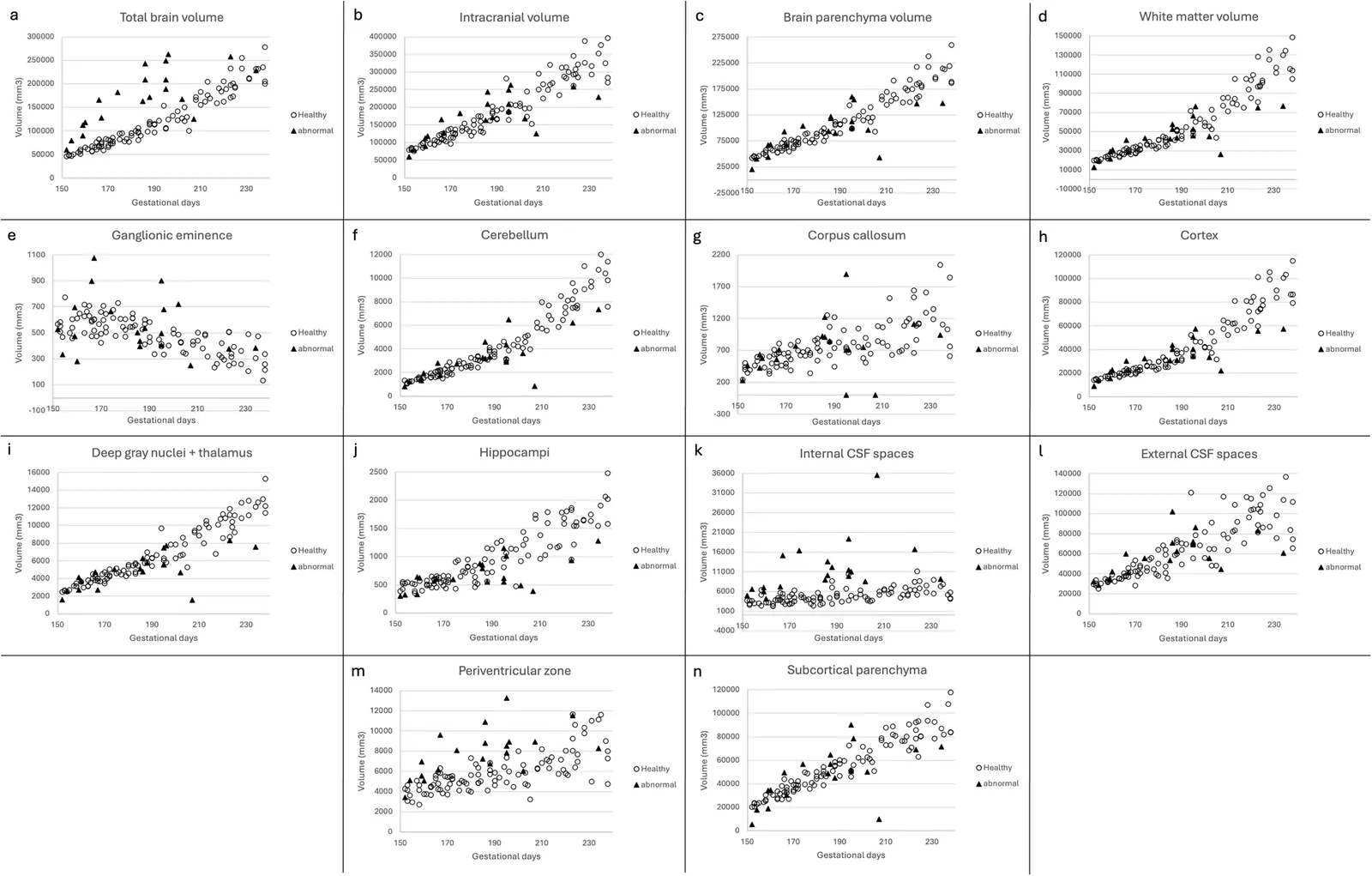
Volumetric assessment of fetal brain structures. Scatterplots depicting volumes (mm^3^) over gestational age (days) calculated from fetal super- resolution MRI: **a** Total brain volume, **b** intracranial volume, **c** brain parenchyma volume, **d** white matter volume, **e** ganglionic eminence, **f** cerebellum, **g** corpus callosum, **h** cortex, **i** deep gray nuclei and thalami, **j** hippocampi, **k** internal cerebrospinal fluid (CSF) spaces, **l** external CSF spaces, **m** periventricular zones (including ventricular and subventricular zones), **n** subcortical parenchyma (including the subcortical layers between but not including the cortical plate and subventricular zone)

Fetuses with and without brain anomalies were examined between 22 to 35 GW. Volumetric assessment of all eleven analyzed brain structures, as well as TBV, BPV, WMV, and ICV, is summarized in Fig. 2 and Table 3. Among the 17 fetuses (20 MRI scans) with subjectively enlarged GE, only 4/17 fetuses (5/20 MRI scans) were found to have GE volumes larger than the reference collective (above 95% confidence interval of the reference collective), while 12/20 had normal GE volumes (within 95% confidence interval) and 3/20 had reduced GE volumes (below 95% confidence interval). Interestingly, most patients (14/17 fetuses, 16/20 MRI scans) had an increased TBV, attributed to ventriculomegaly in most affected patients within this collective (14/17 fetuses, 15/20 MRI scans). Total ICV was only increased in 5/17 fetuses (6/20 MRI scans) in this patient collective, with high rates of ventriculomegaly. Additionally, 8/17 fetuses (8/20 MRI scans) were also found to have enlarged periventricular zones. Depending on the concurrent structural brain anomalies (as indicated in Supplementary Table 1), additional brain structures in patients varied in size, e.g., in the setting of callosal agenesis and cerebellar hypoplasia. Analysis of correlation between z-scores of the reference collective and the group with subjectively enlarged GE showed no correlation between the GE and other analyzed brain structures, nor with TBV, ICV, WMV, or BPV (Supplementary Table 2).

**Table 3:** Volumetric assessment of fetal brain structures among the group with subjectively enlarged ganglionic eminence

Brain structure	Decreased in size	Within 95% confidence interval (normal size)	Enlarged
Brainstem	4 (20%)	13 (65%)	3 (15%)
Corpus callosum	3 (15%)	15 (75%)	2 (10%)
Cerebellum	4 (20%)	13 (65%)	3 (15%)
Cortex	5 (25%)	10 (50%)	5 (25%)
Deep gray nuclei + thalami	5 (25%)	15 (75%)	0 (0%)
External CSF spaces	2 (10%)	16 (80%)	2 (10%)
Ganglionic eminence	3 (15%)	12 (60%)	5 (25%)
Hippocampi	4 (20%)	16 (80%)	0 (0%)
Inner CSF spaces	0 (0%)	5 (25%)	15 (75%)
Periventricular zone	0 (0%)	12 (60%)	8 (40%)
Subcortical parenchyma	5 (25%)	10 (50%)	5 (25%)
Intracranial volume (ICV)	3 (15%)	11 (55%)	6 (30%)
Total brain volume (TBV)	0 (0%)	4 (20%)	16 (80%)
Brain parenchyma volume (BPV)	7 (35%)	9 (45%)	4 (20%)
White matter volume (WMV)	5 (25%)	11 (55%)	4 (20%)
Total	54 (16.9%)	184 (57.5%)	82 (25.6%)

Values provided as the number of cases (percentage of the group)

Sample size *n* = 20

Regarding the comparison of two- and three-dimensional fetal head assessment, a significant correlation of the fronto-occipital and biparietal diameters of the fetal brain and skull with TBV and ICV, respectively, was found (Supplementary Table 3). This confirms the comparability of two- and three-dimensional measurements regarding overall brain and head size.

## Discussion

**Fig. 3:**
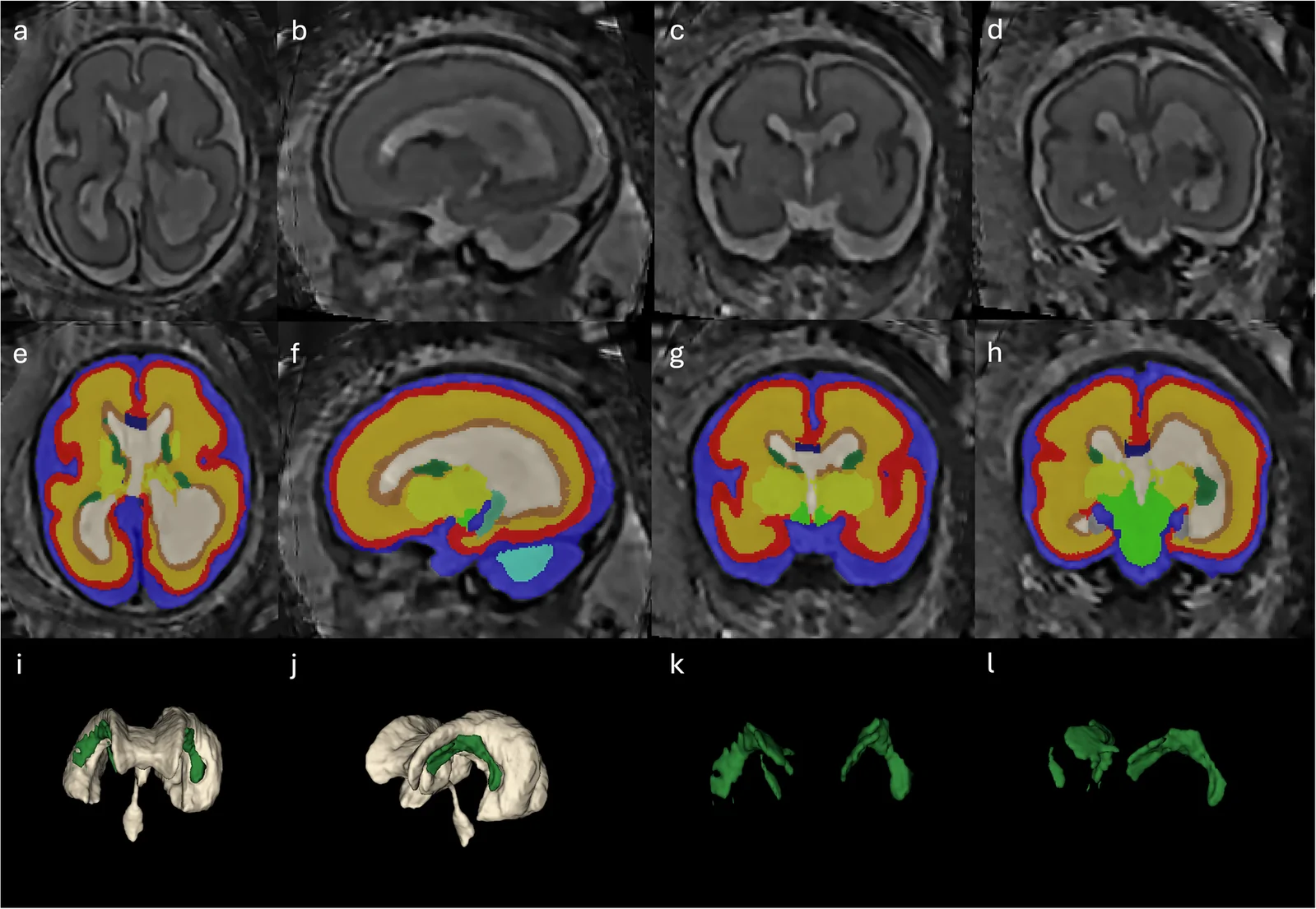
MRI super-resolution reconstruction and tissue segmentation of a patient with subjectively enlarged ganglionic eminence (GE) without confirmation of GE hyperplasia following three-dimensional MRI volumetry and concurrent ventriculomegaly. This figure illustrates the mismatch between subjective visual expert assessment of GE size and quantitative assessment utilizing three-dimensional volumetry. T2-weighted MRI super-resolution reconstruction of a fetus at 24 + 6 gestational weeks in the axial (**a**), paramedian sagittal (**b**), and coronal plane (**c**, **d**). Super-resolution reconstruction in the coronal plane with tissue segmentations in the axial (**e**), paramedian sagittal (**f**), and coronal plane (**g**, **h**). Three-dimensional reconstruction of ventricular system and ganglionic eminence segmentations in a.p. view (**i**) and oblique anterolateral (**j**). Three-dimensional reconstruction of ganglionic eminence segmentations in a.p. view (**k**) and oblique anterolateral (**l**). Color coding: navy blue—outer cerebrospinal fluid (CSF) spaces, red—cortex, orange— subcortical parenchyma (including the subcortical layers between but not including the cortical plate and subventricular zone), yellow—deep gray nuclei and thalamus, brown—periventricular zone, dark blue— corpus callosum, gray—hippocampus, dark green—ganglionic eminence, turquoise—cerebellum, light green—brainstem, white—inner CSF spaces/ventricles

**Fig. 4:**
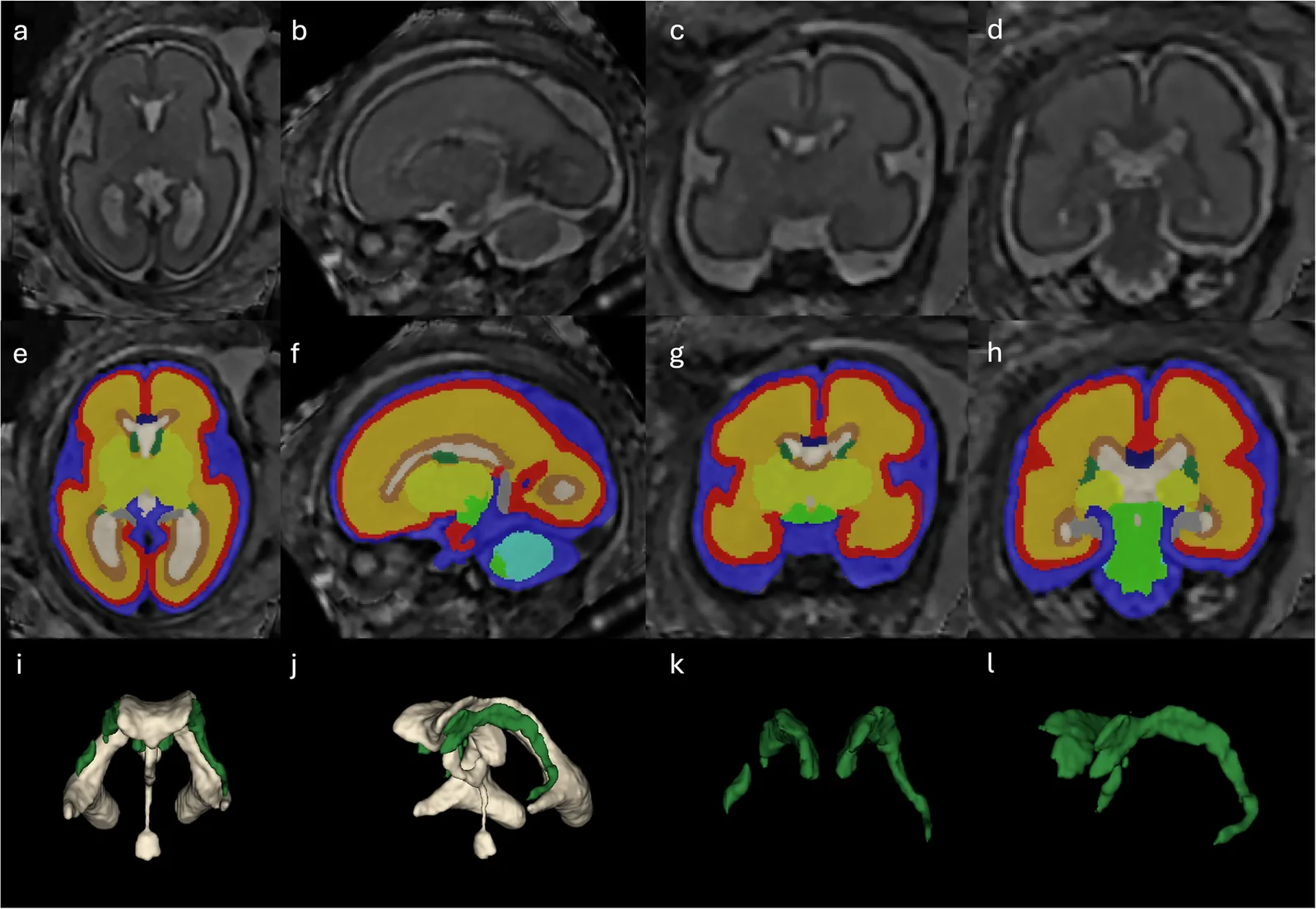
MRI super-resolution reconstruction and tissue segmentation of a control patient. This figure illustrates normal ganglionic eminence size in a fetus (age-matched to the patient in Fig. 3). T2-weighted MRI super-resolution reconstruction of a fetus at 25 + 0 gestational weeks in the axial (**a**), paramedian sagittal (**b**), and coronal plane (**c**, **d**). Super-resolution reconstruction in the coronal plane with tissue segmentations in the axial (**e**), paramedian sagittal (**f**), and coronal plane (**g**, **h**). Three-dimensional reconstruction of ventricular system and ganglionic eminence segmentations in a.p. view (**i**) and oblique anterolateral (**j**). Three-dimensional reconstruction of ganglionic eminence segmentations in a.p. view (**k**) and oblique anterolateral (**l**). Color coding: navy blue—outer cerebrospinal fluid (CSF) spaces, red—cortex, orange— subcortical parenchyma (including the subcortical layers between but not including the cortical plate and subventricular zone), yellow—deep gray nuclei and thalamus, brown—periventricular zone, dark blue— corpus callosum, gray—hippocampus, dark green—ganglionic eminence, turquoise—cerebellum, light green—brainstem, white—inner CSF spaces/ventricles

This study followed the concept of imaging-based deep phenotyping in prenatal medicine and applied super-resolution MRI to quantify transient anatomical substructures of structurally abnormal fetal brains. Deep phenotyping includes genetic analysis and advanced imaging techniques and allows for earlier detection of genetic disorders even at prenatal stages [26, 27]. Volumetric assessment was performed for patients with subjectively enlarged GE and compared to a reference collective. Against the expert rater assessment, GE hyperplasia could only be confirmed in one-fourth of the cases. The change in size relations of the surrounding structures and TBV seems to be the main source of difficulty in visual assessment of the GE in patients with structural CNS anomalies. Specifically, the high occurrence of ventriculomegaly in this collective reflects the importance of the configuration and volume of the ventricular system to subjectively assess the GE, as it is immediately adjacent to the internal CSF spaces and may result in variations in the GE’s configuration (Figs. 3 and  4). Despite the exclusive assessment of cases with subjectively enlarged GE in this cohort, similar findings could be expected in cases of GE hypoplasia based on visual expert assessment alone, although this would require confirmation in future studies. These findings suggest the need for volumetric assessment of the GE based on three-dimensional measurements, particularly in patients with structural CNS anomalies.

**Fig. 5:**
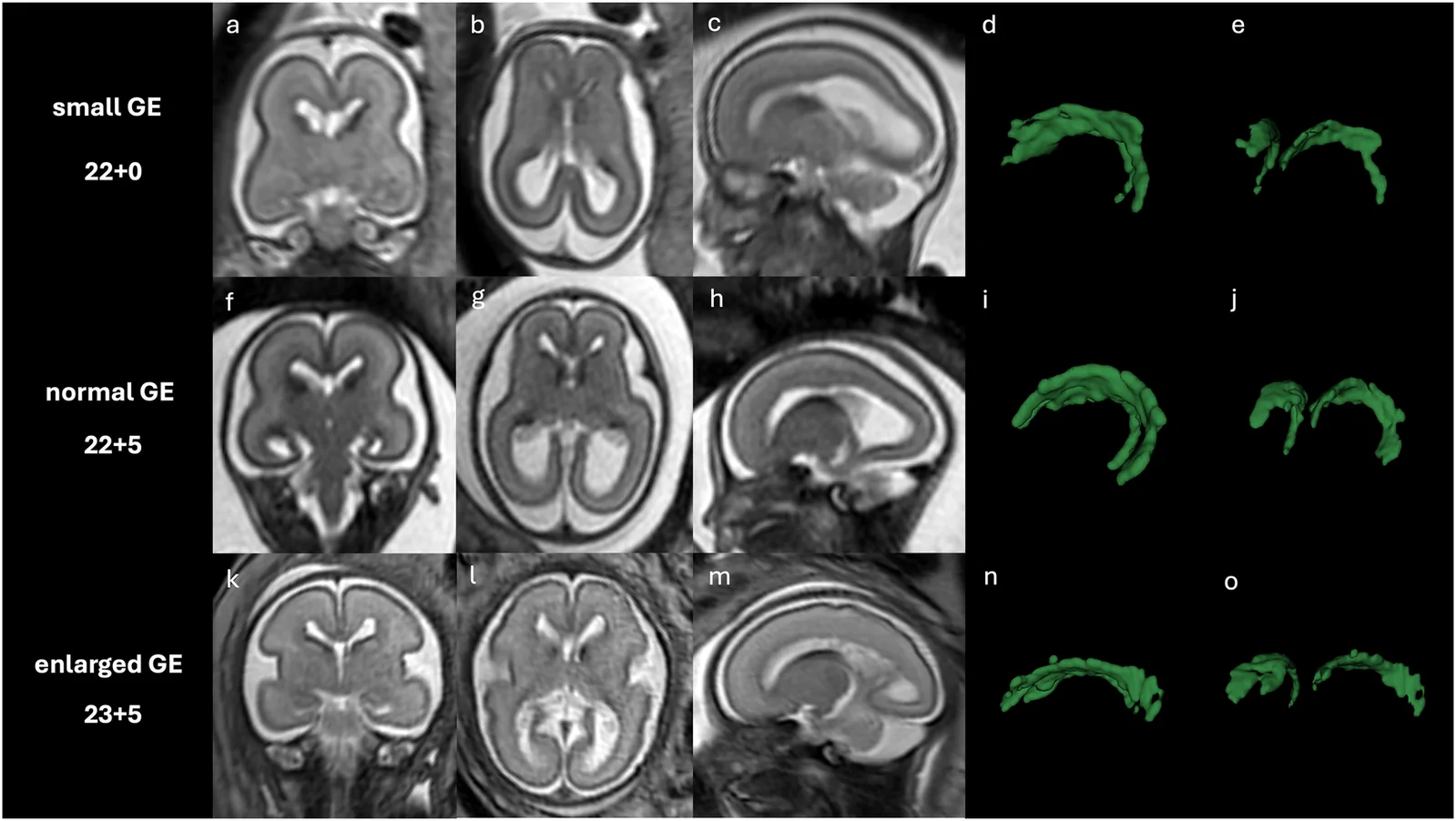
MRI super-resolution reconstruction and three-dimensional reconstruction depict the difficulty of subjective assessment of the ganglionic eminence. Too small ganglionic eminence (GE): T2-weighted MRI super-resolution reconstruction of a fetus at 22 + 0 gestational weeks in the coronal plane (**a**), axial (**b**), and paramedian sagittal plane (**c**). Three-dimensional reconstruction of the ganglionic eminence (GE) in lateral (**d**) and anterolateral oblique view (**e**). Normal-sized GE: T2-weighted MRI super-resolution reconstruction of a fetus at 22 + 5 gestational weeks in the coronal plane (**f**), axial (**g**), and paramedian sagittal plane (**h**). Three-dimensional reconstruction of the ganglionic eminence (GE) in lateral (**i**) and anterolateral oblique view (**j**). Enlarged GE: T2-weighted MRI super-resolution reconstruction of a fetus at 23 + 5 gestational weeks in the coronal plane (**k**), axial (**l**), and paramedian sagittal plane (**m**). Three-dimensional reconstruction of the ganglionic eminence (GE) in lateral (**n**) and anterolateral oblique view (**o**)

Recently, spatiotemporally resolved volumetric reference indices—fetal brain atlases—have been developed for prenatal neurosonography and fetal MRI [28, 29]. Due to limitations of brain growth assessment based on 2D measurements, 3D segmentations of fetal brain substructures promise to provide a higher sensitivity to subtle deviations from normal brain development (Fig. 5). Fetal brain atlases allow for grasping the dynamic changes in size and shape of structures like the GE and will ultimately replace subjective clinical evaluations [7, 8, 17, 18]. Currently, super-resolution MRI and semi-automated manual segmentation of the GE can be a time-consuming task in clinical practice. In the near future, machine learning-based algorithms may replace manual segmentations and provide reliable and robust automated volumetry of the GE. Ultimately, MRI or US-based GE volumetry may be used as a clinical problem-solving tool to overcome subjective biases and misclassifications in imaging-based deep phenotyping.

Imaging-based deep phenotyping allows a deeper understanding of the underlying pathophysiological processes involved in disease manifestations:

One exemplary case in this study (22 + 5 GW) presented with vermian hypo- and dysplasia, dysplastic tectum, malrotated hippocampi, malpositioned upper and lower limbs, cleft jaw and palate and double outlet right ventricle. Genetic work-up included whole-exon sequencing of the fetus and both parents, identifying a de novo mutation of the FGFR2 gene associated with Apert syndrome. The observed structural anomalies match this genetic diagnosis. Interestingly, the GE volume showed no deviation from the normal developmental trajectory despite the affection of some target structures of progenitor cells from the GE. Additional ex vivo histological studies are necessary to confirm the lack of GE involvement in Apert syndrome, but were unavailable due to a lack of parental consent following termination of pregnancy.

Another exemplary case (29 + 4 GW) presented with microcephaly, abnormal lamination, complete callosal agenesis, hypo- and dysplastic brainstem, cerebellar hypoplasia, vermian dysplasia, and intraventricular hemorrhage. Following volumetry of the GE, we found a significant decrease in GE volume compared to the reference collective (below 95% confidence interval). With GE volumes being relatively too low, the number of potential progenitor cells arising in the GE was also decreased, presumably substantially impacting its migrational target structures.

Assessment of GE size in isolation may be insufficient for diagnosis; interpretation should be integrated within the overall imaging and clinical context. As previous studies have shown, volumetric changes, specifically enlargement/persistence of the GE, were found to be associated with various co-occurring structural anomalies, including macrocephaly, abnormal lamination, polymicrogyria (e.g., tubulinopathies (e.g., TUBA1A) and ARX gene-related disorders), hemimegalencephaly (e.g., PI3K-AKT-MTOR pathway diseases), and mitochondrial disorders, highlighting the clinical relevance of GE evaluation [8, 12, 17, 30]. Among the 4 patients (5 MRI scans) with volumetric verification of the hypertrophic appearance of the GE, the following co-occurring anomalies with known association with abnormal GE size development were identified: macrocephaly (2 cases), abnormal lamination (e.g., subependymal heterotopia/polymicrogyria, 3 cases), and lissencephaly (1 case). Additional findings were megacisterna magna (1 case), ventriculomegaly (3 cases), complete callosal agenesis (1 case), and Probst bundles (1 case).

Structural anomalies within the GE may vary greatly based on various etiologies. Many studies have investigated defects, including hemorrhagic and cystic anomalies within the GE [7, 8, 17, 30]. However, not only structural defects and hypoplasia may result in persistent clinical consequences: Hyperplasia of the GE has been shown to be associated with macrocephaly [8]. This may appear counterintuitive, as an increase in GE volume based on cell retention should result in a reduction of migration of progenitor cells towards target structures, including the cortex, thus resulting in cortical hypoplasia. Other (patho-)physiological processes, including axonal pruning, might also be involved specifically in the setting of macrocephaly, resulting in increased TBV. Overall, enlargement is thus an important structural anomaly of the GE, despite the difficulties of quantifying this transient fetal brain structure. Interestingly, we found no correlation of GE and TBV in this collective, which is in contrast to the aforementioned study [8], potentially due to the wide spectrum of concurrent CNS anomalies in the investigated cohort. Regardless, the lack of correlation of GE volume to TBV or ICV— and consequently fetal head size—highlights the necessity of selective volumetric assessment of the GE.

### Clinical implications

The demonstrated unreliability of subjective evaluation of the GE necessitates its quantitative volumetric assessment during fetal MRI. This is particularly important in patients with structural CNS anomalies, which could further hinder quantification based exclusively on visual assessment, as is the current clinical practice. Our contribution is to emphasize that expert assessment alone may be misleading in certain circumstances, particularly in the presence of other structural abnormalities. Identification of GE anomalies may allow for earlier detection of structural defects within their migrational targets and associated anomalies, including cortical malformations [8]. Detection of GE anomalies should urge counseling physicians to consider additional genetic testing, including tubulinopathies, ARX gene-related disorders, PI3K-AKT-MTOR pathway diseases, and mitochondrial disorders, when appropriate, in light of the observed co-occurring structural anomalies.

### Research implications

Future research should explore larger patient collectives and include postnatal outcome data. Due to the recent inclusion of patients, complete analysis of postnatal outcomes cannot be provided in this study, but is ongoing.

### Strengths and limitations

We present the first study examining GE enlargement based on super-resolution fetal MRI volumetry, providing precise, in vivo quantification of this transient fetal brain structure. The investigated patients all presented with GE enlargement without cavitations, cysts and hemorrhages and were compared to a large reference collective without confounding comorbidities. Segmentation data were of high accuracy and reproducibility as indicated by the strong inter- and intrarater correlations.

The histological subregions, including medial, lateral, and caudal GE, were not subdivided during segmentation of the investigated patients due to the small size of this transient fetal brain structure, limited resolution, and the lack of histological correlation, which was not available given the in vivo nature of this study. Despite the relatively small size of the GE compared to other investigated fetal brain structures and a more scattered appearance of volumetric results, the high degree of intra- and interrater agreement in the investigated subset of patients indicates a high level of accuracy and reproducibility of the measurements. Due to the retrospective nature of this study, complete follow-up data, including genetic work-up and postnatal clinical and imaging-based correlation, were not available for all patients analyzed. Due to restrictions from our IRB, patients with selected bodily structural anomalies were included in the control group of this study. To our knowledge, the selected anomalies have no confounding effect on fetal neurodevelopment. Furthermore, the heterogeneity of included anomalies outweighs the limited selection bias in this study.

## Conclusion

Expert visual assessment and 2D fetal brain biometry were insufficient to accurately characterize fetal brain anomalies, particularly regarding GE size. Especially if the fetal brain anatomy is grossly distorted—such as in ventriculomegaly—the correct size or volume estimations may be inconclusive or misleading. The results of this study highlight the added value of volumetric quantification of the GE in fetuses with concurrent brain malformations, providing an unbiased, objective, reproducible, and valid quantitative tool for imaging-based deep phenotyping of CNS anomalies in the fetal brain.

## Supplementary information

Supplementary information

## Abbreviations

BPV: Brain parenchyma volume
CSF: Cerebrospinal fluid
GE: Ganglionic eminence
GWs: Gestational weeks
ICV: Intracranial volume
TBV: Total brain volume
WMV: White matter volume

## References

[1] Stiles J, Jernigan TL (2010) The basics of brain development. Neuropsychol Rev 20:327–348. 10.1007/s11065-010-9148-4 21042938 PMC2989000

[2] Wichterle H, Turnbull DH, Nery S, Fishell G, Alvarez-Buylla A (2001) In utero fate mapping reveals distinct migratory pathways and fates of neurons born in the mammalian basal forebrain. Development 128:3759–3771. 10.1242/dev.128.19.3759 11585802

[3] Nery S, Fishell G, Corbin JG (2002) The caudal ganglionic eminence is a source of distinct cortical and subcortical cell populations. Nat Neurosci 5:1279–1287. 10.1038/nn971 12411960

[4] Kepecs A, Fishell G (2014) Interneuron cell types are fit to function. Nature 505:318–326. 10.1038/nature12983 24429630 PMC4349583

[5] Petilla Interneuron Nomenclature Group, Ascoli GA, Alonso-Nanclares L et al (2008) Petilla terminology: nomenclature of features of GABAergic interneurons of the cerebral cortex. Nat Rev Neurosci 9:557–568. 10.1038/nrn2402 18568015 PMC2868386

[6] Bozzi Y, Casarosa S, Caleo M (2012) Epilepsy as a neurodevelopmental disorder. Front Psychiatry 3:19. 10.3389/fpsyt.2012.00019 22457654 PMC3306997

[7] Righini A, Frassoni C, Inverardi F et al (2013) Bilateral cavitations of ganglionic eminence: a fetal MR imaging sign of halted brain development. AJNR Am J Neuroradiol 34:1841–1845. 10.3174/ajnr.A3508 23598830 PMC7965643

[8] Scarabello M, Righini A, Severino M et al (2021) Ganglionic eminence anomalies and coexisting cerebral developmental anomalies on fetal mr imaging: multicenter-based review of 60 cases. AJNR Am J Neuroradiol 42:1151–1156. 10.3174/ajnr.A7062 33707279 PMC8191678

[9] Gilani A, Hove JLV, Thomas JA, Kleinschmidt-DeMasters BK (2020) Distinguishing encephaloclastic lesions resulting from primary or secondary pyruvate dehydrogenase deficiency from other neonatal or infantile cavitary brain lesions. Pediatr Dev Pathol 23:189–196. 10.1177/1093526619876448 31542992

[10] Malik R, Pai EL-L, Rubin AN et al (2019) Tsc1 represses parvalbumin expression and fast-spiking properties in somatostatin lineage cortical interneurons. Nat Commun 10:4994. 10.1038/s41467-019-12962-4 31676823 PMC6825152

[11] Hang J-F, Hsu C-Y, Lin S-C et al (2017) Thyroid transcription factor-1 distinguishes subependymal giant cell astrocytoma from its mimics and supports its cell origin from the progenitor cells in the medial ganglionic eminence. Mod Pathol 30:318–328. 10.1038/modpathol.2016.205 27910945

[12] Pogledic I, Mankad K, Severino M et al (2024) Prenatal assessment of brain malformations on neuroimaging: an expert panel review. Brain 147:3982–4002. 10.1093/brain/awae253 39054600 PMC11730443

[13] Tan AP, Svrckova P, Cowan F, Chong WK, Mankad K (2018) Intracranial hemorrhage in neonates: a review of etiologies, patterns and predicted clinical outcomes. Eur J Paediatr Neurol 22:690–717. 10.1016/j.ejpn.2018.04.008 29731328

[14] Ulfig N (2002) Ganglionic eminence of the human fetal brain-new vistas. Anat Rec 267:191–195. 10.1002/ar.10104 12115267

[15] Altmann R, Rechberger T, Altmann C et al (2023) Development of the prosencephalic structures, ganglionic eminence, basal ganglia and thalamus at 11 + 3 to 13 + 6 gestational weeks on 3D transvaginal ultrasound including normative data. Brain Struct Funct 228:2089–2101. 10.1007/s00429-023-02679-y 37712966 PMC10587255

[16] Contro E, Volpe N, Larcher L et al (2023) Normal and abnormal appearance of fetal ganglionic eminence on second-trimester three-dimensional ultrasound. Ultrasound Obstet Gynecol 62:398–404. 10.1002/uog.26229 37099497

[17] Righini A, Cesaretti C, Conte G et al (2016) Expanding the spectrum of human ganglionic eminence region anomalies on fetal magnetic resonance imaging. Neuroradiology 58:293–300. 10.1007/s00234-015-1622-5 26608601

[18] Stuempflen M, Taymourtash A, Kienast P et al (2023) Ganglionic eminence: volumetric assessment of transient brain structure utilizing fetal magnetic resonance imaging. Ultrasound Obstet Gynecol 62:405–413. 10.1002/uog.26232 37099530

[19] Prayer D, Malinger G, De Catte L et al (2023) ISUOG Practice Guidelines (updated): performance of fetal magnetic resonance imaging. Ultrasound Obstet Gynecol 61:278–287. 10.1002/uog.26129 36722431 PMC10107509

[20] Kyriakopoulou V, Vatansever D, Davidson A et al (2017) Normative biometry of the fetal brain using magnetic resonance imaging. Brain Struct Funct 222:2295–2307. 10.1007/s00429-016-1342-6 27885428 PMC5504265

[21] Gholipour A, Rollins CK, Velasco-Annis C et al (2017) A normative spatiotemporal MRI atlas of the fetal brain for automatic segmentation and analysis of early brain growth. Sci Rep 7:476. 10.1038/s41598-017-00525-w 28352082 PMC5428658

[22] Ebner M, Wang G, Li W et al (2020) An automated framework for localization, segmentation and super-resolution reconstruction of fetal brain MRI. Neuroimage 206:116324. 10.1016/j.neuroimage.2019.116324 31704293 PMC7103783

[23] Ciceri T, Squarcina L, Pigoni A et al (2023) Geometric reliability of super-resolution reconstructed images from clinical fetal MRI in the second trimester. Neuroinformatics 21:549–563. 10.1007/s12021-023-09635-5 37284977 PMC10406722

[24] Pier DB, Gholipour A, Afacan O et al (2016) 3D super-resolution motion-corrected MRI: validation of fetal posterior fossa measurements. J Neuroimaging 26:539–544. 10.1111/jon.12342 26990618 PMC4983257

[25] Yushkevich PA, Piven J, Hazlett HC et al (2006) User-guided 3D active contour segmentation of anatomical structures: significantly improved efficiency and reliability. Neuroimage 31:1116–1128. 10.1016/j.neuroimage.2006.01.015 16545965

[26] Dhombres F, Morgan P, Chaudhari BP et al (2022) Prenatal phenotyping: a community effort to enhance the Human Phenotype Ontology. Am J Med Genet C Semin Med Genet 190:231–242. 10.1002/ajmg.c.31989 35872606 PMC9588534

[27] Paquette AG, Hood L, Price ND, Sadovsky Y (2020) Deep phenotyping during pregnancy for predictive and preventive medicine. Sci Transl Med 12:eaay1059. 10.1126/scitranslmed.aay1059 31969484 PMC7268934

[28] Namburete AIL, Papież BW, Fernandes M et al (2023) Normative spatiotemporal fetal brain maturation with satisfactory development at 2 years. Nature 623:106–114. 10.1038/s41586-023-06630-3 37880365 PMC10620088

[29] Makropoulos A, Counsell SJ, Rueckert D (2018) A review on automatic fetal and neonatal brain MRI segmentation. Neuroimage 170:231–248. 10.1016/j.neuroimage.2017.06.074 28666878

[30] Goergen SK, Alibrahim E, Christie J et al (2021) The fetus with ganglionic eminence abnormality: head size and extracranial sonographic findings predict genetic diagnoses and postnatal outcomes. AJNR Am J Neuroradiol 42:1528–1534. 10.3174/ajnr.A7131 33958329 PMC8367629

