# Supplementary material for: What you see is not always what you get—MRI-based ganglionic eminence volumetry challenges subjective assessment in CNS anomalies
Source: Eur Radiol. 2026 May 21;36(10):7395–405. doi: 10.1007/s00330-026-12636-y (PMC13574848; doi:10.1007/s00330-026-12636-y)
Supplement: Supplementary file 1 [file 330_2026_12636_MOESM1_ESM.pdf]

What you see is not always what you get - MRI-Based Ganglionic Eminence Volumetry Challenges Subjective Assessment in CNS Anomalies

ELECTRONIC SUPPLEMENTARY MATERIAL

Eur Radiol (2026) Stuempflen M, Kienast P, Schmidbauer VU, et al.

Supplementary Table 1: Overview of patients with subjectively enlarged ganglionic eminence

Subjectively enlarged ganglionic eminence group

Gestational age	Sex	# MRIs	Final diagnosis after MRI
21+5	female		cerebral hypoplasia, callosal thickening, abnormal lamination, delayed gyration, hypoplastic brainstem, cerebellar hypoplasia, vermian hypoplasia, unremarkable whole exon sequencing
22+0	male	*	mild symmetrical ventriculomegaly, delayed opercularisation, delayed gyrification with frontooccipital gradient, irregular ependyma, mild cerebellar hypoplasia, suspected lissencephaly
22+5	female		mild asymmetrical ventriculomegaly, irregular borders of lateral ventricles and ventricular zones, delayed calcarine sulcus formation
22+5	male		vermian hypo- and dysplasia, dysplastic tectum, malrotated hippocampi, malpositioned upper and lower limbs, cleft jaw and palate, vitium cordis (DORV), de novo FGFR2-mutation, Apert syndrome, later interuterine fetal demise
22+6	male		gastroschisis
23+5	female	*	megacisterna magna, subependymal heterotopia, suspected bilateral polymicrogyria in ventral region and parietal, macrocephaly
23+6	male		fumarat hydratase deficiency syndrome, lissencephaly type II, diffuse polymicrogyria, delayed gyration, moderate ventriculomegaly, macrocephaly
24+6	female		severe asymmetrical ventriculomegaly, subependymal heterotopia, occipital dysgyria, bilateral schizencephal clefts, megacisterna magna
26+3	female		mild asymmetrical ventriculomegaly
26+4	male		hemimegencephaly of right hemisphere, left hemisphere normal, maternal COVID infection during gestation
26+4	male		delayed gyration, periventricular cysts, signal alterations in white matter, macrocephaly
26+6	male	*	lissencephaly type I, delayed gyration, abnormal lamination, abnormal gyrification, subependymal heterotopia, mild ventriculomegaly
27+6	female		delayed gyration + opercularisation, malpositioned hippocampi, borderline ventriculomegaly, widened external CSF spaces, vermian hypoplasia, hydronephrosis
27+6	male		moderate bilateral ventriculomegaly, complete commissural agenesis, Probst bundles, malroted hippocampi
27+6	male		delayed opercularisation, bilateral frontal polymicrogyria, abnormal lamination

28+0	female	*	megacisterna magna, subependymal heterotopia
28+6	female	*	mild asymmetrical ventriculomegaly, delayed lamination
29+4	female		hypo-/dysplastic brainstem, cerebellar hypoplasia, vermian dysplasia, abnormal lamination, decreased cerebral thickness, complete callosal agenesis, small intraventricular hemorrhage
31+6	male		suspected ciliopathy, callosal hypoplasia, malrotated hippocampi, thin chiasma, congenital pulmonary airway malformation, hydronephrosis, double kidney, shortened limbs
33+3	female	*	borderline ventriculomegaly, delayed gyration, signal alterations in frontal periventricular zone

* patient with two MRI scans

Control group

Gestational age	Sex	# MRIs	Final diagnosis after MRI
21+5	male		hydronephrosis
21+6	female	*	congenital pulmonary airway malformation
21+6	male		jejunal atresia
21+6	male		placenta accreta
22+0	male		club feet
22+1	male		double kidney
22+3	female	*	subpulmonal sequester
22+4	female		myoma
22+4	male		polycystic dysplastic kidney
22+4	female		premature rupture of membranes
22+6	male	*	lymphangioma leg/sacral region
23+0	female		pelvic kidney
23+2	male		congenital diaphragmatic hernia
23+2	male		no structural anomaly

23+2	female		ureterocele left
23+3	female		cleft lip
23+4	female		congenital pulmonary airway malformation
23+4	male		no structural anomaly
23+5	male		midface anomaly, hypertelorism
23+5	male		no structural anomaly
23+5	male		no structural anomaly
23+6	male		cleft lip
23+6	female		gallbladder elongated
23+6	male		herniation into cord
24+0	male		no structural anomaly
24+1	male		no structural anomaly
24+2	male		no structural anomaly
24+2	male		no structural anomaly
24+2	male		oligohydramnios
24+3	male		no structural anomaly
24+3	male		oligohydramnios
24+4	male		cleft lip + palate, abnormal positioning of right lower extremity
24+4	male		no structural anomaly
24+4	male	*	premature rupture of membranes
25+0	male		no structural anomaly
25+0	male		premature rupture of membranes, single umbilical artery
25+1	male		kidney agenesis
25+2	male		gallbladder elongated
25+3	female		pelvic kidney
25+4	female		diaphragmal eventration, lung hypoplasia
25+5	male		gallbladder elongated

25+5	male	*	placenta praevia
25+5	male		premature rupture of membranes
26+0	female		no structural anomaly
26+1	female		amelia of hand
26+1	male		dysplastic kidney
26+1	male		gallbladder dilated, peristent right umbilical vein
26+1	male		premature rupture of membranes
26+2	male		gastroschisis
26+5	male		no structural anomaly
26+6	male		premature rupture of membranes
26+6	male		signal alterations in liver after transfusion, rhesus incompatibility
26+6	female		splenic cyst
27+1	female	*	gallbladder agenesis
27+1	male		no structural anomaly
27+1	female		premature rupture of membranes
27+3	female	*	subpulmonal sequester
27+5	male		no structural anomaly
27+6	male		no structural anomaly
27+6	female		premature rupture of membranes
28+0	female		no structural anomaly
28+2	female		no structural anomaly
28+4	male		no structural anomaly
28+4	male		polyhydramnios
28+6	male		no structural anomaly
29+0	male		club feet
29+0	male		congenital pulmonary airway malformation
29+1	female		no structural anomaly

29+2	male		midface hypoplasia, coanal atresia, exophthalmus
29+5	male	*	premature rupture of membranes
29+5	male		congenital pulmonary airway malformation
30+0	male		no structural anomaly
30+0	male		posterior urethral valves, multicystic kidneys, oligohydramnion
30+2	male		pelvic kidney
30+3	female		ovarian cyst
30+3	male		polyhydramnios
30+4	male		no structural anomaly
31+0	male		jejunal atresia, microcolon
31+1	male		liver cyst
31+2	male		no structural anomaly
31+3	female		no structural anomaly
31+3	male		no structural anomaly
31+4	female		no structural anomaly
31+6	female		no structural anomaly
31+6	male		no structural anomaly
31+6	female		placenta biparta
32+0	female	*	gallbladder agenesis
32+0	male		no structural anomaly
32+1	female		no structural anomaly
32+1	male		no structural anomaly
32+4	female		esophageal atresia
32+4	female		ovarian torsion
33+0	female	*	congenital pulmonary airway malformation
33+0	male		no structural anomaly
33+3	female		ovarian cyst

33+4	female		ovarian cyst
33+6	male	*	placenta praevia
34+0	male	*	lymphangioma leg/sacral region
34+0	male		enlarged thymus
34+0	female		no structural anomaly

* patient with two MRI scans

Supplementary Table 2: Correlation of z-values of volumetric fetal brain measurements from patients with subjectively enlarged ganglionic eminence and the control group (95% confidence interval).

		Brain parenchyma volume (BPW)	Brainstem	Corpus callosum	Cerebellum	Cortex	Deep gray nuclei	External CSF spaces	Ganglionic eminence	Hippocampi	Inner CSF	Intracranial volume (ICV)	Peri-ventricular zone	Subcortical parenchyma	Total brain volume (TBV)	White matter volume (WMV)
**Brain parenchyma volume (BPW)**	**Pearson correlation**	1	0.857**	0.609**	0.872**	0.962**	0.907**	0.721**	0.171	0.776**	-0.180	0.957**	0.459*	0.979**	0.953**	0.964**
	**Significance (2-sided)**		0.001	0.004	0.001	0.001	0.001	0.001	0.471	0.001	0.447	0.001	0.042	0.001	0.001	0.001
**Brainstem**	**Pearson correlation**	0.857**	1	0.438	0.885**	0.894**	0.875**	0.774**	0.046	0.731**	-0.312	0.868**	0.377	0.794**	0.876**	0.920**
	**Significance (2-sided)**	0.001		0.054	0.001	0.001	0.001	0.001	0.846	0.001	0.181	0.001	0.102	0.001	0.001	0.001
**Corpus callosum**	**Pearson correlation**	0.609**	0.438	1	0.562**	0.536*	0.522*	0.433	-0.175	0.613**	-0.306	0.563**	0.076	0.636**	0.558*	0.542*
	**Significance (2-sided)**	0.004	0.054		0.010	0.015	0.018	0.056	0.461	0.004	0.190	0.010	0.750	0.003	0.011	0.014
**Cerebellum**	**Pearson correlation**	0.872**	0.885**	0.562**	1	0.913**	0.815**	0.799**	0.129	0.723**	-0.356	0.886**	0.258	0.825**	0.885**	0.929**
	**Significance (2-sided)**	0.001	0.001	0.010		0.001	0.001	0.001	0.588	0.001	0.124	0.001	0.272	0.001	0.001	0.001
**Cortex**	**Pearson correlation**	0.962**	0.894**	0.536*	0.913**	1	0.880**	0.804**	0.097	0.802**	-0.184	0.967**	0.454*	0.894**	0.974**	0.996**
	**Significance (2-sided)**	0.001	0.001	0.015	0.001		0.001	0.001	0.685	0.001	0.437	0.001	0.045	0.001	0.001	0.001
**Deep gray nuclei**	**Pearson correlation**	0.907**	0.875**	0.522*	0.815**	0.880**	1	0.602**	0.028	0.745**	-0.415	0.826**	0.229	0.888**	0.831**	0.910**
	**Significance (2-sided)**	0.001	0.001	0.018	0.001	0.001		0.005	0.906	0.001	0.069	0.001	0.331	0.001	0.001	0.001
**External CSF spaces**	**Pearson correlation**	0.721**	0.774**	0.433	0.799**	0.804**	0.602**	1	0.0711	0.512*	-0.084	0.879**	0.454*	0.642**	0.879**	0.795**
	**Significance (2-sided)**	0.001	0.001	0.056	0.001	0.001	0.005		0.766	0.0211	0.725	0.001	0.044	0.002	0.001	0.001
**Ganglionic eminence**	**Pearson correlation**	0.171	0.046	-0.175	0.129	0.097	0.028	0.071	1	-0.176	0.135	0.163	0.378	0.190	0.144	0.085
	**Significance (2-sided)**	0.471	0.846	0.461	0.588	0.685	0.906	0.766		0.458	0.570	0.492	0.100	0.422	0.545	0.722
**Hippocampi**	**Pearson correlation**	0.776**	0.731**	0.613**	0.723**	0.802**	0.745**	0.512*	-0.176	1	-0.348	0.712**	0.274	0.717**	0.727**	0.806**
	**Significance (2-sided)**	0.001	0.001	0.004	0.001	0.001	0.001	0.021	0.458		0.133	0.001	0.243	0.001	0.001	0.001
**Inner CSF spaces**	**Pearson correlation**	-0.180	-0.312	-0.306	-0.356	-0.184	-0.415	-0.084	0.135	-0.348	1	-0.064	0.508*	-0.206	-0.073	-0.241
	**Significance (2-sided)**	0.447	0.181	0.190	0.124	0.437	0.069	0.725	0.570	0.133		0.790	0.022	0.383	0.759	0.305
**Intracranial volume (ICV)**	**Pearson correlation**	0.957**	0.868**	0.563**	0.886**	0.967**	0.826**	0.879**	0.163	0.712**	-0.064	1	0.545*	0.905**	0.998**	0.959**
	**Significance (2-sided)**	0.001	0.001	0.010	0.001	0.001	0.001	0.001	0.492	0.001	0.790		0.013	0.001	0.001	0.001
**Periventricular zone**	**Pearson correlation**	0.459*	0.377	0.076	0.258	0.454*	0.229	0.454*	0.378	0.274	0.508*	0.545*	1	0.375	0.536*	0.414
	**Significance (2-sided)**	0.042	0.102	0.750	0.272	0.045	0.331	0.044	0.100	0.243	0.022	0.0130		0.104	0.015	0.069
**Subcortical parenchyma**	**Pearson correlation**	0.979**	0.794**	0.636**	0.825**	0.894**	0.888**	0.642**	0.190	0.717**	-0.206	0.905**	0.375	1	0.892**	0.901**
	**Significance (2-sided)**	0.001	0.001	0.003	0.001	0.001	0.001	0.002	0.422	0.001	0.383	0.001	0.104		0.001	0.001
**Total brain volume (TBV)**	**Pearson correlation**	0.953**	0.876**	0.558*	0.885**	0.974**	0.831**	0.879**	0.144	0.727**	-0.073	0.998**	0.536*	0.892**	1	0.966**
	**Significance (2-sided)**	0.001	0.001	0.011	0.001	0.001	0.001	0.001	0.545	0.001	0.759	0.001	0.015	0.001		0.001
**White matter volume (WMV)**	**Pearson correlation**	0.964**	0.920**	0.542*	0.929**	0.996**	0.910**	0.795**	0.085	0.806**	-0.241	0.959**	0.414	0.901**	0.966**	1
	**Significance (2-sided)**	0.001	0.001	0.014	0.001	0.001	0.001	0.001	0.722	0.001	0.305	0.001	0.069	0.001	0.001	

** The correlation is significant on the level of 0.01 (two-sided).

* The correlation is significant on the level of 0.05 (two-sided).

Sample size N = 20

Supplementary Table 3: Correlation of two-dimensional and three-dimensional volumetric measurements of overall fetal brain and skull size

		Brain biparietal diameter	Brain fronto-occipital diameter	Skull biparietal diameter	Skull fronto-occipital diameter	Total brain volume	Intracranial volume
**Brain biparietal diameter**	**Spearman-Rho correlation coefficient**	1	0.804**	0.918**	0.743**	0.757**	0.736**
	**Significance (2-sided)**		<0.001	<0.001	<0.001	<0.001	<0.001
**Brain fronto-occipital diameter**	**Spearman-Rho correlation coefficient**	0.804**	1	0.832**	0.790**	0.753**	0.738**
	**Significance (2-sided)**	<0.001		<0.001	<0.001	<0.001	<0.001
**Skull biparietal diameter**	**Spearman-Rho correlation coefficient**	0.918**	0.832**	1	0.835**	0.734**	0.710**
	**Significance (2-sided)**	<0.001	<0.001		<0.001	<0.001	<0.001
**Skull fronto-occipital diameter**	**Spearman-Rho correlation coefficient**	0.743**	0.790**	0.835**	1	0.646**	0.606**
	**Significance (2-sided)**	<0.001	<0.001	<0.001		0.002	0.005
**Total brain volume**	**Spearman-Rho correlation coefficient**	0.757**	0.753**	0.734**	0.646**	1	0.775**
	**Significance (2-sided)**	<0.001	<0.001	<0.001	0.002		<0.001
**Intracranial volume**	**Spearman-Rho correlation coefficient**	0.736**	0.738**	0.710**	0.606**	0.775**	1
	**Significance (2-sided)**	<0.001	<0.001	<0.001	0.005	<0.001	

** The correlation is significant on the level of 0.01 (two-sided).
